# Graphene Oxide and Carbon Nanotubes-Based Polyvinylidene Fluoride Membrane for Highly Increased Water Treatment

**DOI:** 10.3390/nano11102498

**Published:** 2021-09-24

**Authors:** Jungryeong Chae, Taeuk Lim, Hao Cheng, Jie Hu, Sunghoon Kim, Wonsuk Jung

**Affiliations:** 1 School of Mechanical Engineering, Chungnam National University, Daejeon 34134, Korea; wndfud486@naver.com (J.C.); taewook9409@g.cnu.ac.kr (T.L.); chenghao@g.cnu.ac.kr (H.C.); h387669019@gmail.com (J.H.); 2 Department of Electronics Convergence Engineering, Wonkwang University, Iksan 54538, Korea

**Keywords:** graphene oxide, carbon nanotubes, polyvinylidene fluoride, water treatment, surface morphology

## Abstract

As contaminated water increases due to environmental pollution, the need for excellent water treatment is increased, and several studies have reported the polyvinylidene fluoride (PVDF)-based water treatment membranes. However, the PVDF membrane has several problems such as low filtration performance, fouling resistance, and difficulty in precisely controlling the morphology of the pores and hydrophilicity. Therefore, we newly produced a water treatment PVDF membrane containing graphene oxide (GO) and multi-walled carbon nanotubes (MWCNTs) to improve the filtration performance. Surface properties of the fabricated membrane such as morphology, and size of pores, hydrophilicity, and water flux of the membrane were investigated. Additionally, the performance of these membrane filters was evaluated for free residual chlorine, turbidity, chromaticity, magnesium, sulfate, and particulates class 1 according to drinking water management act criteria, respectively. A performance improvement of at least 108.37% was observed compared to the Pure PVDF filter module and anti-fouling effects due to the functional groups of GO and MWCNTs. These results reveal that proposed membrane can accelerate the development of various water filtration applications.

## 1. Introduction

An imbalance in the supply and demand for water is emerging due to the deterioration of water quality caused by rapid urbanization and industrialization around the world. As the amounts and variety of contaminants increase, it is impossible to remove contaminants using conventional water purification methods [1]. To solve this problem, the concept of advanced water treatment was introduced instead of the existing water treatment processes (flocculation → precipitation → sand filtration → disinfection) in developed countries. Advanced water treatment is a process used to remove organic pollutants and pathogenic microorganisms that are not completely removed by general water treatment and includes ozone treatment technology, activated carbon treatment technology, the advanced oxidation process (AOP), and membrane filtration technology [2,3]. Among them, the membrane filtration method has the advantages of high processing efficiency and easy maintenance through a single process and removing suspended solids over a certain size. It is used for water purification, wastewater treatment, and seawater desalination; membranes are classified according to pore size and are used for various types of materials to be removed [4,5,6]. The membrane material used in filtration process can use all polymers, but it is quite limited in practical applications due to differences in physicochemical properties [7,8].

PVDF has been used as a membrane material for water treatment because it has excellent chemical resistance, heat resistance, and mechanical properties [9,10]. Marshall et al. [11] reported the useful properties and applications of PVDF, and how it can be made into films, and finally review the limitations of the chemical and thermal stability of PVDF. Additionally, Wu et al. [12], Malakootian et al. [13] and Chen et al. [14] have reported studies to remove 1,1,1-trichloroethane, magnesium, and turbidity using PVDF membranes, respectively. While PVDF membranes are widely used in wastewater filtration, radiation changes are potentially dangerous to PVDF membranes. Lee et al. [15] observed that exposure to UV radiation caused cracks and fractures on the surface of PVDF membranes, which resulted in reduced tensile strength. Electron beam (EB) radiation has been shown to increase the internal cross-linking of PVDF [16,17] and consequently reduce the size of pores in PVDF membranes, and the degree of pore uniformity is greatly reduced upon exposure to radiation. In addition, radiation-induced changes in surface roughness and surface functionalization reduced the water flux in the membrane [18]. γ -radiation has been similarly demonstrated for causing chain breaks in the cross-linked structure [19]. However, due to its strong hydrophobic properties, organic matter is adsorbed on the surface of the membrane and has the drawback of blocking pores, resulting in a rapid decrease in transmittance [20,21]. To solve this phenomenon, many studies have been conducted to change the existing hydrophobic properties to hydrophilic properties [22,23,24]. Kim et al. [22] used a mixture of oxygen and methane gas for plasma treatment of commercial PVDF membranes and found 62% C=O groups and 38% C-O groups by core-level spectral analysis fitting, with a large number of carbonyl groups causing the PVDF surface to change from hydrophobic to hydrophilic. Zhao et al. [24] obtained PVDF membranes by immersing PVDF membranes in TMC n-hexane solution after plasma treatment with a plasma device, and the analysis showed that the number of hydrophilic polar groups on the membrane surface increased.

Recently, there has been a trend of increasing research on a method of improving membrane performance by grafting various nanomaterials, such as graphene oxide (GO), multiwalled carbon nanotubes (MWCNTs), zeolites, and nanoparticles, with a polymer [25,26,27,28]. In the case of GO, various oxygen functional groups, such as carbonyl groups (C=O), carboxyl groups (–COOH), and hydroxyl groups (–OH), are combined so that the dispersion is stable in aqueous solutions and organic solvents. Additionally, since it is possible to develop a variety of properties that polymer materials do not have when used with other polymer materials, such studies are ongoing [29,30,31].

Among various nano materials, MWCNTs have characteristics such as high flexibility, low mass density, high porosity, and antibacterial properties, so they are applied in various fields, such as sensors and batteries. However, due to van der Waals forces, the dispersibility in organic solvents and aqueous solutions is very low, and the bundling phenomenon is strong. Therefore, a method of improving dispersibility, such as the introduction of functional groups on the surface of MWCNTs, has been studied [32,33,34,35]. As such, various studies are currently underway on a separation membrane for water treatment in which GO and MWCNTs are added to improve the existing separation membrane characteristics and filtration performance [36,37].

However, there are still many problems to be solved regarding fouling resistance and filtration performance in using the PVDF membrane [38,39]. In addition, the PVDF membrane is difficult to control the size and number of pores precisely. Additionally, there has been little research on whether the filter performance of the PVDF membrane meets the drinking water criteria.

Therefore, in this paper, we newly suggested a fabrication method of a water treatment GO/MWCNTs/PVDF (GMP) membrane. According to the content ratio of GO and MWCNTs, the number and size of pores on the membrane surface can be precisely controlled. Additionally, we characterized the properties of the membrane such as water flux, detection of GO and CNTs in the elution, surface properties such as the number and size of pores, and water contact angle measurement, respectively. We also investigated the excellent filtration performance for free residual chlorine, turbidity, chromaticity, magnesium, sulfate, and particulates class 1 according to drinking water management act criteria. As a result, we made in-depth investigation regarding the phenomena related to carbon material-based PVDF membrane for water treatment.

## 2. Experiments and Method

### 2.1. Materials and Fabrication Process of GMP Membrane

**Figure 1:**
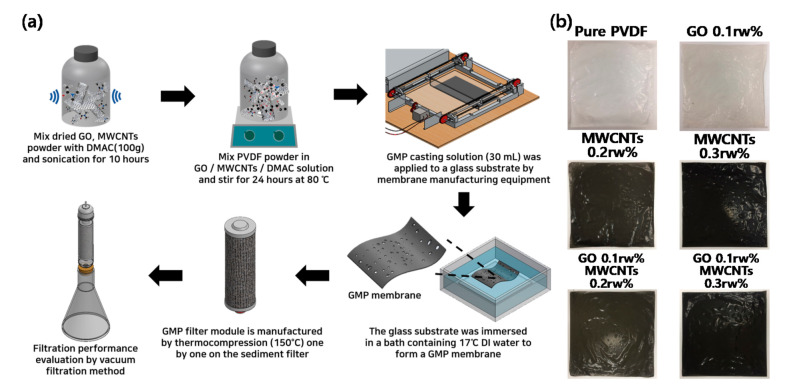
(**a**) The process schematic of GMP membranes and (**b**) digital photograph of the membrane by condition.

GMP membranes were synthesized based on non-solvent-induced phase separation (NIPS) phenomena. Several synthesized ratio conditions were selected to analyze the effectiveness of GO and MWCNTs against the pure PVDF membrane as shown in Table S1. This fabrication process is shown in Figure 1. GO (GO V-20, purchased from Standard Graphene, Ulsan, Korea), MWCNTs (CM-130, purchased from Hanwah Chemical, Pangyo-ro, Korea) and PVDF (purchased from Dongguan East Plastic Trade, Dongguan, China) were dried in an oven at 80 °C for 24 h according to their respective composition ratios. The chemical composition and detailed material properties of GO and CNTs used in these experiments are referred to supporting information. After mixing the dried GO and MWCNTs with dimethylacetamide (DMAC; purity 99.5% purchased from Daejung Chemicals and Metals, Siheung-si, Korea), they were dispersed for 10 h through sonication (40 kHz). The dispersed GO/MWCNTs/DMAC solution was mixed with PVDF and then mixed through mechanical stirring at 80 °C for 24 h, and 2 h of sonication (40 kHz) was conducted to synthesize a casting solution. The synthesized GMP casting solution (30 mL) was applied to a glass substrate by pushing it with a steel blade through the membrane manufacturing equipment, as in Figure 1. The glass substrate was immersed in a bath (50 (W) × 50 (D) × 30 (H)) containing 17 ℃ DI water to introduce a phase transition reaction. The casting solution reacted with DI water to cause solidification, and the residual solution was removed to form a membrane. The manufactured membrane was stored in DI water at 20 °C. The membrane for each condition was fabricated in the same way. The thickness of each membrane was the same as the 0.12 mm by the membrane manufacturing 3D-printed equipment, which uses a 24 V DC motor, made in the laboratory to fabricate the accurate thickness of each membrane. The fabricated membrane for each condition is shown in Figure 1b, and it can be seen that the opacity increases as the carbon nano material increases, respectively. The company of each material used in this fabrication process is described in detail in supporting contents.

### 2.2. Production of GMP Filter Module

To test the filtration performance using the drinking water management act criteria, a module was manufactured in the form of a water purifier filter (Figure S1). A sediment filter (40 (W) × 40 (D) × 150 (H) mm) was used as the frame of the filter. The manufactured GMP membrane was cut to 150 × 150 mm according to the size of the sediment filter. The cut membranes were fixed through thermocompression (150 °C) one by one on the sediment filter. The membrane thickness was 96 mm and fixed once more with nonwoven fabric. After that, it was manufactured in a structure in which filtered water can be obtained only through the filter by capping the top and bottom of the filter module using hot melt. The completed filter module was placed in a filter housing (L: 300 mm) to test the filtration performance. However, only visible particles can be removed by the sediment filter used in these experiments, and it is not possible to remove the water treatment reagents.

## 3. Results and Discussions

### 3.1. Characterization of GMP Membranes

**Figure 2:**
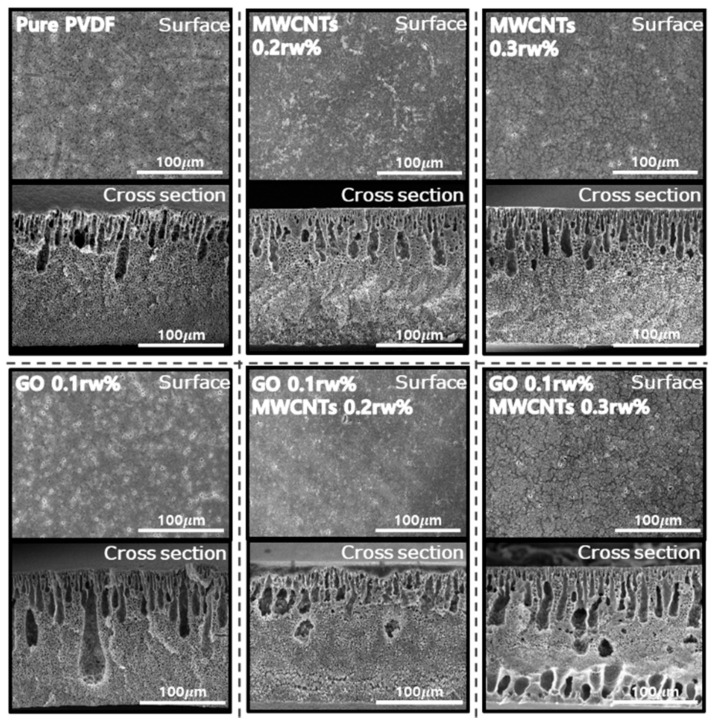
FE-SEM images of the bottom surface and cross-section for condition.

We investigated the surface, cross-section, and internal structure of the membrane using field-emission scanning electron microscopy (FE-SEM, Hitachi S-4800, Tokyo, Japan) as shown in Figure 2. Characteristics of pores on the surface and cross-section of the membrane change according to the addition of GO and MWCNTs.

Since the surface shown in the SEM image coagulates in the phase transfer process, these pores are created. Additionally, pores on the front and rear surfaces were present in an asymmetric structure when the PVDF membrane was manufactured through the NIPS method [40]. Increasing the amount of GO rw%, the FE-SEM image showed that the number of surface pores was higher than that of Pure PVDF. On the other hand, as the amount of the MWCNTs increased, FE-SEM images showed that the number of surface pores decreased, but the size of the pores increased. Additionally, when GO is added, the pore is much deeper and wider than the Pure PVDF pore. The membrane with the ratios of GO 0.1 rw%/MWCNTs 0.3 rw% showed a larger number and size of pores compared to the membrane with GO 0.1 rw%/MWCNTs 0.2 rw, respectively. In addition, the sample showed even pore distribution on both the top and bottom surfaces of the membrane at that condition.

**Figure 3:**
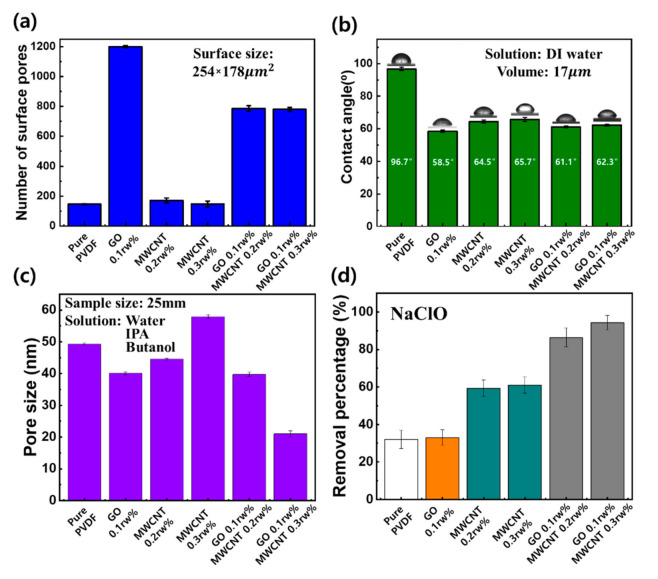
(**a**) Number of surface pores, (**b**) water contact angle, (**c**) pore size distribution, and (**d**) removal percentage of free residual chlorine (NaClO) with a single membrane sheet.

To analyze the number of surface pores at each condition, SEM images per 100 µm unit area were analyzed using the Image J program, as shown in Figure 3a. In the case of pure PVDF, the number of pores was 146 ± 3.0 per unit area. Additionally, the number of pores of 1199 ± 7.5 for GO 0.1 rw%, 170 ± 17.5 for MWCNTs 0.2 rw%, 148 ± 19.5 for MWCNTs 0.3 rw%, 785 ± 19.1 for GO 0.1 rw%/MWCNTs 0.2 rw%, and 780 ± 12.5 for GO 0.1 rw%/MWCNTs 0.3 rw% were measured, respectively. This number of pores of GO was almost five times more than MWCNTs. Additionally, the number of pores decreased as the content of MWCNTs increased because pores are formed based on the phase transition process. This phase transition process is based on the oxygen functional groups of GO, which cause dispersion between DI water molecules (nonsolvent) and DMAC (solvent), resulting in a rapid phase change. In the case of MWCNTs, the phase transition speed is slower than that of GO, so the number of pores decreases. Additionally, we verified that GO and MWCNTs were uniformly distributed within the PVDF membrane through TEM images as shown in Figure S2. Through these results, the structure and the number of pores of the membrane can be controlled by the addition of GO and MWCNTs to pure PVDF.

Water contact angle is one of the important factors affecting water flux. The more hydrophilic the surface of membrane is, the more solution inflow into the membrane filter increases. Therefore, the water contact angle was also measured to analyze surface properties of whether hydrophilic and hydrophobic, and the results are shown in Figure 3b. Water contact angles of membrane for each condition were measured five times, respectively.

The hydrophobic properties of pure PVDF were confirmed with a contact angle of 96.7° ± 1.08°. However, as GO and MWCNTs were added, the contact angles decreased by at least 32%. The contact angles of each membrane were 58.5 ± 0.80 for GO 0.1 rw%, 64.5 ± 0.99 for MWCNTs 0.2 rw%, 65.7 ± 1.25 for MWCNTs 0.3 rw%, 61.1 ± 0.60 for GO 0.1 rw%/MWCNTs 0.2 rw%, and 62.3 ± 0.50 for GO 0.1 rw%/MWCNTs 0.3 rw%, respectively. The surface properties of membranes were changed to be hydrophilic due to the oxygen functional groups of GO and hydrophilic functional groups (amines) of MWCNTs during the phase transition process.

Additionally, we analyzed the pore size of each membrane using the liquid–liquid porometer (LLP) method based on the capillary flow as shown in Figure 3c. LLP is a method used to measure the pore size by calculating the pressure discharged from the pores, which is capable of measuring the pore size from 2 nm to several tens of um at low pressure using liquid–liquid with low interfacial tension. The membrane with a size of Ø25 mm was pretreated by filtering Galwick solution (Sigma-Aldrih, St. Louis, MO, USA) (15.9 dyne/cm^2^) by vacuum filtration to reduce the surface tension, and analysis was performed using DI water, IPA, and butanol. Nitrogen gas was used to apply pressure to the DI water that pushes IPA and butanol into the pores. The average pore sizes were 49.3 ± 0.28 nm for Pure PVDF, 40.1 ± 0.41 nm for GO 0.1 rw%, 44.6 ± 0.23 nm for MWCNTs 0.2 rw%, 57.9 ± 0.66 nm for MWCNTs 0.3 rw%, 39.8 ± 0.64 nm for GO 0.1 rw%/MWCNTs 0.2 rw%, and 21.0 ± 0.98 nm for GO 0.1 rw%/MWCNTs 0.3 rw%, respectively. The pore-size distribution of the MWCNTs was larger than that of GO, and it was confirmed that the pore size was reduced when GO was added. Therefore, pore sizes can be controlled by the addition of GO and MWCNTs to pure PVDF.

In order to evaluate the material removal performance of each membrane, we analyzed the filtering results for various substances such as free residual chlorine (NaClO), turbidity, and chromaticity, respectively, which are essential criteria of the drinking water quality control act in Korea. In addition, filtering performance was verified for ionic material and particular class 1, which were referenced to US criteria. Ionic material filtration criteria were verified using inorganic (magnesium) and organic (sulfate ion) materials. Among the various filtering tests, we first validated the removal performance analysis on NaClO, as shown in Figure 3d. Each membrane is made of the same size and thickness, and the membrane conditions are pure PVDF, GO 0.1 rw%, MWCNTs 0.1, 0.2, 0.3%, and a mixture of various MWCNTs with GO 0.1 rw%, respectively.

Each membrane was cut into Ø49 mm pieces, and 250 mL of solution with an influent concentration of 2.5 ppm was filtered by vacuum filtration. The concentration of NaClO before and after filtration was measured five times using a portable drinking water colorimeter. The removal percentages of NaClO were measured as follows: 32.0 ± 4.9% for Pure PVDF, 33.1 ± 4.2% for GO 0.1 rw%, 42.9 ± 1.5%, 59.3 ± 4.5%, 61.0 ± 4.4% for MWCNTs of 0.1, 0.2, 0.3 rw%, and 65.4 ± 4.0%, 86.4 ± 4.5%, 94.4 ± 3.9% for MWCNTs of 0.1, 0.2, 0.3 rw% with GO 0.1 rw%, respectively, as shown in Figure 3d.

In the case of pure PVDF membrane, it showed a certain level of NaClO removal rate, which is analyzed to be the effect of nanoscale pores on the surface. In addition, as the MWCNTs content increases from 0.1 to 0.3 rw%, the rate of NaClO elimination was observed to increase, as shown in Figure 3d. This phenomenon is attributed to the superior adsorption function of the functional group. However, if only GO is added to the pure PVDF, only a 1.1% increase in NaClO removal rate can be seen. Additionally, if GO is added to the MWCNTs samples, it can be seen that filtering performance is better than MWCNTs alone, as shown in Figure 3d.

As shown above, it was confirmed that GO 0.1 rw%/MWCNTs 0.3 rw% was the most suitable condition to remove NaClO as a water treatment membrane. Therefore, the filter module was manufactured under the conditions of GO 0.1 rw%/MWCNTs 0.3 rw%, and filter performance was verified through comparative analysis with the Pure PVDF filter module under various conditions. Additionally, we compared commercialized water purifier filters with GO 0.1 rw%/MWCNT 0.3 rw% filters for water permeability comparison, as shown in Figure S3. Sodium hypochlorite (NaClO) was diluted with DI Water and tested with a concentration of 2.5 ppm. The material properties of ClO- are referred to supporting information. The filtration solution with a 250 mL was tested at 0.8 bar by the vacuum filtration method, and the pre- and post- filtration time was measured three times with a portable multi-item water quality meter. Each commercial water filter used in the experiment was a sedimentation filter (49 mm × 33 cm, Pore size: 10 μm), and an ultra-filter (49 mm × 33 cm, pore size: 5 μm), respectively. However, commercial filters showed a low removal rate of NaClO in spite of their fast filtration time of 11.2 ± 2.14 s with the sediment filter, 33.94 ± 1.54 s with the ultra-filter, respectively. GMP filters showed 100% removal performance measured even at lower pressure, while this filter had less water permeability than the commercial filter, as shown in Figure S3. Additionally, the analysis was conducted with an ultraviolet-visible spectrophotometer to measure whether GO and MWCNTs are eluted or not. The GO dispersion solution was prepared by mixing 50 mL of DI Water with 0.1 rw% of GO using 1 h of sonication. The MWCNT dispersant was mixed with 15 mg of MWCNT and 0.5 g of Surfactant (SDS) in 50 mL of DI water for 3 h of sonication. Afterwards, the dispersant was manufactured at 10,000 rpm through centrifugation for 3 h. As a measurement sample, GO 0.1 rw%/MWCNT 0.3 rw% single separation film and GO 0.1 rw%/MWCNT 0.3 rw% filter were used, and the experiment was conducted a total of five times per sample by filtrating 1 L of DI Water. As a result of the measurement, the peak value was measured at a wavelength of 215.08 nm at GO and MWCNT solution. However, no peak value was detected for GO 0.1 rw%/MWCNT 0.3 rw% separation film and filter. Therefore, no elution phenomenon was generated for GO, MWCNT, and it was possible to confirm that GO and MWCNT remained stable in the structure.

### 3.2. GMP Filter Module Filtration Performance

To evaluate the filtering performance of the GMP (GO 0.1 rw%/MWCNTs 0.3 rw%) filter module, we perform a comparative analysis on the pure PVDF module such as free residual chlorine, turbidity, chromaticity, magnesium, sulfate, and particulates class 1 tests. The vacuum filtration method using the aspirator (EYELRA) was used to verify the performance of the filter under operating pressure of 0.8 bar. Each filtration solution was made in 1 L according to the drinking water quality process test standard, and performance evaluation was performed five times for the same filter.

**Figure 4:**
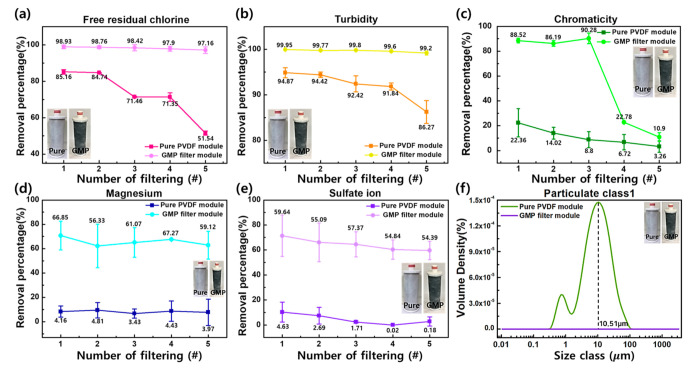
Comparative analysis of pure PVDF module and GMP module for (**a**) free residual chlorine, (**b**) turbidity, (**c**) chromaticity, (**d**) magnesium, (**e**) sulfate ion, and (**f**) particulate class1, respectively.

Each numerical value of free residual chlorine, turbidity, and chromaticity were measured using a portable drinking water colorimeter. Magnesium and sulfate ions levels were measured using coupled plasma atomic emission spectroscopy (ICP-AES), and ion chromatography, respectively. Additionally, articulates class 1 was measured using a particle size analyzer, as shown in Figure 4. To test free residual chlorine, solutions of dilute sodium hypochlorite solution (NaClO) were prepared with DI water to make solution concentrations greater than 2.0 ppm. For the turbidity test, undiluted solutions were diluted with DI water to achieve a solution concentration greater than 20 NTU. As a result of the measurement, the free residual chlorine and species causing turbidity were removed more than 97% for the GMP module, even after five filtration processes, as shown in Figure 4a,b. However, the pure PVDF module showed 85.2% and 94.9% removal percentage of NaClO and turbidity at the first filtration, respectively. Even in the third filtration, the removal level showed a sharp decrease and after the fifth filtration, it was lower than 51.5% for NaClO and 86.3% for turbidity, respectively. To measure chromaticity levels, a solution concentration was higher than 40 Pt/Co, which was diluted with DI water (Figure 4c). In the case of the chromaticity, the GMP module had a removal rate of 86% or more by the third time, but filtration performance was reduced to 10% thereafter. In the case of the Pure PVDF filter module, it was possible to remove up to 22%, but it later decreased to 3%. For the magnesium and sulfate removal tests, the standard solutions were diluted with DI water to make solution concentrations of more than 20 ppm and 400 ppm, respectively. In the case of GMP module, the magnesium and sulfate ions were removed by at least 54% even after five times of filtration, as shown in Figure 4d,e. However, the Pure PVDF filter module was only able to remove up to 4%, which means that any removal was hardly observed. The filtration rate of our prepared membrane was 98.93% for free residual chlorine. Wu et al. [12] achieved 97% filtration rate for 1,1,1-trichloroethane (TCA) in PVDF membrane using LiCl-H2O as an additive. In addition, Xu et al. [29] used titanate nanofiber membranes (TNF) for the separation of direct yellow (DY) dye solution and the removal rate was 55.8%, while the maximum removal rate for chromaticity was 90.28% for our prepared membrane. The removal of magnesium from water was experimented by Malakootian et al. [13] using a commercial nano-filtration membrane and the removal rate of magnesium was 62.26% and on the other hand, in the case of our experiment, it reached 70.74%. The removal rate of turbidity by ultrafiltration membrane PVDF was 99.12% [14], compared to our superior result of 99.95%.

Particulates Class 1 are the range of particle sizes that can be removed by using dust powder mixed with particles of various sizes based on the water quality management standard of the National Sanitation Foundation (NSF) in the United States of America. The standard solution of the class 1 particulate was made by mixing 600 mL of DI water with 0.09 g of dust powder and the five consecutive filtration experiments were subsequently conducted using this standard solution. As shown in Figure 4f, the pure PVDF filter module was unable to remove 100% of the 0.01–3282 µm particles of the standard solution, with the highest volume density of 10.51 µm. On the other hand, the GMP filter module showed 100% removal performance after particle class 1 filtration test and thus the particle distribution was measured at 0%, as shown in Figure 4f.

Through the evaluation of the six factors of filtration performance, it was confirmed that the Pure PVDF filter module has little ability in removing mineral elements and ionic substances. In addition, the filtration performance has been shown to decrease significantly as the number of filtrations repeats, which is attributed to fouling phenomena.

In the case of the GMP filter module to which carbon nanomaterials were added, it could be confirmed that most of the six elements were excellently removed due to the functionalities of GO, MWCNTs and the nanopore size. Even though, the fouling phenomenon also occurs in the GMP filter module in the case of high-concentration acid solutions such as the chromatic solution, the effect of the fouling phenomenon has emerged very slowly compared to the pure PVDF filter modules.

## 4. Conclusions

In summary, we newly fabricated a water treatment GMP membrane having GO/MWCNTs based on the PVDF. The number and size of pores on the surface of GMP membrane were precisely controlled according to the content ratio of GO and MWCNTs. Additionally, we conducted characterization of GMP membrane such as water flux, detection of GO and CNTs in the elution, and analysis of the structure of GO/CNTs/PVDF by TEM and SEM images compared to pure PVDF membrane, respectively.

The performance of these GMP membrane filters was evaluated for free residual chlorine, turbidity, chromaticity, magnesium, sulfate, and particulates class 1 according to drinking water management act criteria. In the case of the Pure PVDF filter module, it was confirmed that it had little ability to remove mineral elements and ionic substances. Moreover, the performance was dramatically decreased as the filtration test repeated. However, in the case of the GMP filter module, it showed excellent filtration performance in all six assessment criteria according to drinking water management act criteria including minerals and ionic substances due to the functional groups of GO and MWCNTs. Especially, this filter had good removal performance despite repeated filtration tests. Therefore, it was confirmed that the introduction of the GO/MWCNT material at an appropriate ratio improves the surface properties and water purification functions of the PVDF membrane. These results reveal that GMP membrane can accelerate the development of various water filtration applications.

## Data Availability

Not applicable.

## Supplementary Materials

The following are available online at https://www.mdpi.com/article/10.3390/nano11102498/s1, Figure S1: GMP Filter Module Manufacturing Process; Table S1: Ratio of Carbon Materials of GMP membranes at each condition; Figure S2: TEM images of (a) pure PVDF and (b–d) GMP membranes; Figure S3: Comparison of filtration time and removal percentages of NaClO, for commercial water filter and GMP filter module, respectively; Figure S4: Analysis of detection of graphene oxide and carbon nanotubes in the elution.

## References

[1] Kummu M., Ward P.J., Moel H.D., Varis I. (2010). Is physical water scarcity a new phenomenon? Global assessment of water shortage over the last two millennia. Environ. Res. Lett..

[2] Kim J.O., Lee K.K. (2006). Treatment of Refractory Dye Wastewater Using AOPs. J. Korean Geo.-Environ. Soc..

[3] Cho K.H., Kim Y.B. (1998). The trend and prospect of advanced water treatment process using ozonizer. Proc. KIIEE Annu. conf..

[4] Geise G.M., Park H.B., Sagle A.C., Freeman B.D., McGrath J.E. (2011). Water permeability and water/salt selectivity tradeoff in polymers for desalination. J. Membr. Sci..

[5] Yu L.Y., Xu Z.L., Shen H.M., Yang H. (2009). Preparation and characterization of PVDF–SiO_2_ composite hollow fiber UF membrane by sol-gel method. J. Membr. Sci..

[6] Lee Y.M., Shim J.K. (1999). Separation Membranes in Water Treatment and their Applications. Polym. Sci. Technol..

[7] Kang J.S., Kim J.P. (2005). Tech-Trend for Membrane in Water and Wastewater Treatment. Polym. Sci. Technol..

[8] Guan Y.F., Huang B.C., Qian C., Wang L.F., Yu H.Q. (2017). Improved PVDF membrane performance by doping extracellular polymeric substances of activated sludge. Water Res..

[9] Zeng G.Y., He Y., Zhan Y.Q., Zhang L., Pan Y., Zhang C.L., Yu Z.X. (2016). Novel polyvinylidene fluoride nanofiltration membrane blended with functionalized halloysite nanotubes for dye and heavy metal ions removal. J. Hazard. Mater..

[10] Tian X.Z., Xue J.F. (2008). Poly (vinylidene fluoride-co-hexafluoropropene) (PVDF-HFP) membranes for ethyl acetate removal from water. J. Hazard. Mater..

[11] Marshall J.E., Zhenova A., Roberts S., Petchey T., Zhu P., Dancer C.E.J., McElroy C.R., Kendrick E., Goodship V. (2021). On the Solubility and Stability of Polyvinylidene Fluoride. Polymers.

[12] Wu B., Tan X., Li K., Teo W.K. (2006). Removal of 1,1,1-trichloroethane from water using a polyvinylidene fluoride hollow fiber membrane module: Vacuum membrane distillation operation. Sep. Purif. Technol..

[13] Malakootian M., Mahvi A.H., Fatehizadeh A., Ehrampoush M.H. (2011). Efficiency of calcium and magnesium removal by nanofiltration membrane from synthetic water under different operating conditions. Toloo-e-Behdasht.

[14] Chen Y., Xu W., Zhu H., Wei D., He F., Wang D., Du B., Wei Q. (2019). Effect of turbidity on micropollutant removal and membrane fouling by MIEX/ultrafiltration hybrid process. Chemosphere.

[15] Lee M.J., Ong C.S., Lau W.J., Ng B.C., Ismail A.F., Lai S.O. (2016). Degradation of PVDF-based composite membrane and its impacts on membrane intrinsic and separation properties. J. Polym. Eng..

[16] Adem E., Rickards J., Burillo G., Avalos-Borja M. (1999). Changes in poly-vinylidene fluoride produced by electron irradiation. Radiat. Phys. Chem..

[17] Nasef M.M., Saidi H., Dahlan K.Z.M. (2002). Investigation of electron irradiation induced-changes in poly(vinylidene fluoride) films. Polym. Degrad. Stab..

[18] Jaleh B., Gavar N., Fakhri P., Muensit N., Taheri S.M. (2015). Characteristics of PVDF membranes irradiated by electron beam. Membranes.

[19] Medeiros A.S., Gual M.R., Pereira C., Faria L.O. (2015). Thermal analysis for study of the gamma radiation effects in poly(vinylidene fluoride). Radiat. Phys. Chem..

[20] Liu F., Hashim N.A., Liu Y., Abed M.M., Li K. (2011). Progress in the production and modification of PVDF membranes. J. Membr. Sci..

[21] Kang G.D., Cao Y.M. (2014). Application and modification of poly (vinylidene fluoride) (PVDF) membranes—A review. J. Membr. Sci..

[22] Kim E.S., Kim Y.J., Yu Q.S., Deng B.L. (2009). Preparation and characterization of polyamide thin-film composite (TFC) membranes on plasma-modified polyvinylidene fluoride (PVDF). J. Membr. Sci..

[23] Chang I.S., Clech P.L., Jefferson B., Judd S. (2002). Membrane Fouling in Membrane Bioreactors for Wastewater Treatment. J. Environ. Eng..

[24] Zhao X.Z., Xuan H.X., Chen Y.L., He C.J. (2015). Preparation and characterization of superior antifouling PVDF membrane with extremely ordered and hydrophilic surface layer. J. Membr. Sci..

[25] Shi F., Ma Y., Ma J., Wang P., Sun W. (2013). Preparation and characterization of PVDF/TiO_2_ hybrid membranes with ionic liquid modified nano-TiO_2_ particles. J. Membr. Sci..

[26] Liang S., Kang Y., Tiraferri A., Giannelis E.P., Huang X., Elimelech M. (2013). Highly Hydrophilic Polyvinylidene Fluoride (PVDF) Ultrafiltration Membranes via P ostfabrication Grafting of Surface-Tailored Silica Nanoparticles. ACS Appl. Mater. Interfaces.

[27] Zhang J., Xu Z., Shan M., Zhou B., Li Y., Li B., Niu J., Qian X. (2013). Synergetic effects of oxidized carbon nanotubes and graphene oxide on fouling control and anti-fouling mechanism of polyvinylidene fluoride ultrafiltration membranes. J. Membr. Sci..

[28] Jamshidifard S., Koushkbaghi S., Hosseini S., Rezaei S., Karamipour A., Irani M. (2019). Incorporation of UiO-66-NH2 MOF into the PAN/chitosan nanofibers for adsorption and membrane filtration of Pb(II), Cd(II) and Cr(VI) ions from aqueous solutions. J. Hazard. Mater..

[29] Xu C., Wang C., He X., Lyu M., Wang S., Wang L. (2017). Processable graphene oxide-embedded titanate nanofiber membranes with improved filtration performance. J. Hazard. Mater..

[30] Alramadhan S.A., Hammud H.H. (2021). Graphene nickel silica supported nanocomposites as an efficient purifier for water treatment. Appl. Nanosci..

[31] Xu Z., Zhang J., Shan M., Li Y., Li B., Niu J., Zhou B., Qian X. (2014). Organosilane-functionalized graphene oxide for enhanced antifouling and mechanical properties of polyvinylidene fluoride ultrafiltration membranes. J. Membr. Sci..

[32] Fazelabdolabadi B., Khodadadi A.A., Sedaghatzadeh M. (2015). Thermal and rheological properties improvement of drilling fluids using functionalized carbon nanotubes. Appl. Nanosci..

[33] Dwivedi P., Vijayakumar R.P. (2018). Synthesis of UMCNOs from MWCNTs and analysis of its structure and properties for wastewater treatment applications. Appl. Nanosci..

[34] Xie P., Lannoy C.F.d., Ma J., Wang Z., Wang S., Li J., Wiesner M.R. (2016). Improved chlorine tolerance of a polyvinyl pyrrolidone-polysulfone membrane enabled by carboxylated carbon nanotubes. Water Res..

[35] Zhao Y.H., Qian Y.L., Zhu B.K., Xu Y.Y. (2008). Modification of porous poly (vinylidene fluoride) membrane using amphiphilic polymers with different structures in phase inversion process. J. Membr. Sci..

[36] Zhao X., Sui K., Wu W., Liang H., Li Y., Wu Z., Xia Y. (2012). Synthesis and properties of amphiphilic block polymer functionalized multi-walled carbon nanotubes and nanocomposites. Compos. Part A Appl. Sci. Manuf..

[37] Guo Z.Y., Yuan X.S., Geng H.Z., Wang L.D., Jing L.C., Gu Z.Z. (2018). High conductive PPY–CNT surface-modified PES membrane with anti-fouling property. Appl. Nanosci..

[38] Drews A. (2010). Membrane fouling in membrane bioreactors-Characterisation, contradictions, cause and cures. J. Membr. Sci..

[39] Li Q., Elimelech M. (2004). Organic Fouling and Chemical Cleaning of Nanofiltration Membranes: Measurements and Mechanism. Environ. Sci. Technol..

[40] Yuan X.T., Xu C.X., Geng H.Z., Ji Q., Wang L., He B., Jiang Y., Kong J., Lia J. (2020). Multifunctional PVDF/CNT/GO mixed matrix membranes for ultrafiltration and fouling detection. J. Hazard. Mater..

